# Supplementary material for: Developing a Low-Cost Device for Estimating Air–Water ΔpCO2 in Coastal Environments
Source: Sensors (Basel). 2025 Jun 4;25(11):3547. doi: 10.3390/s25113547 (PMC12158275; doi:10.3390/s25113547)
Supplement: Supplementary file 1 [file sensors-25-03547-s001.zip › sensors-3600944-SI.pdf]

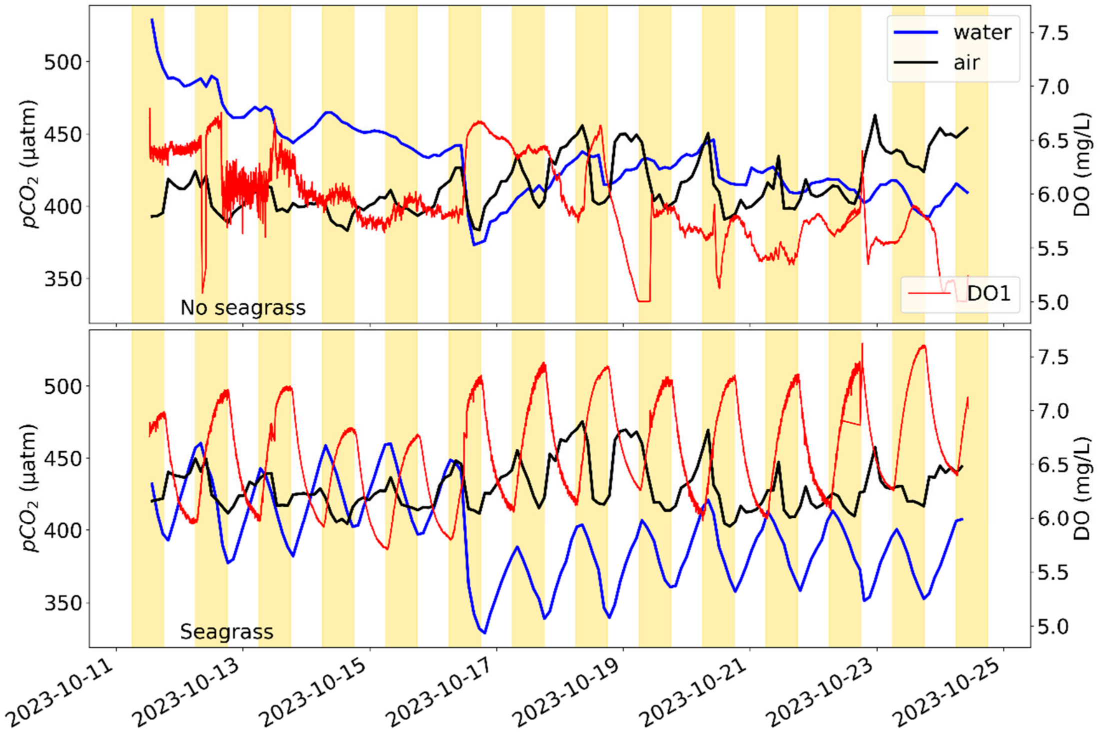

Figure S1. Equilibrated pCO_2 end points plotted for the air (black) and water-side (blue) for the tanks with and without seagrass, as labelled. Yellow background signifies “daytime,” or when the aquarium lights are illuminated, and white background signifies “nighttime,” or when lights are turned off. Dissolved oxygen (DO) is plotted in red.

Calculation S1

<u>Example Calculation for Equations 2-4</u>

$$K30_{CO2} = [(K30_{raw} - K30_{H2O}) * m_{dry}] + b \quad (2)$$

$$K30_{H2O} = (m_{H2O} * V_{H2O} + b_{H2O}) * (H/100) \quad (3)$$

$$V_{H2O} = 6 * 10^{-5}(T^3) + 5 * 10^{-4}(T^2) + 0.055(T) + 0.571 \quad (4)$$

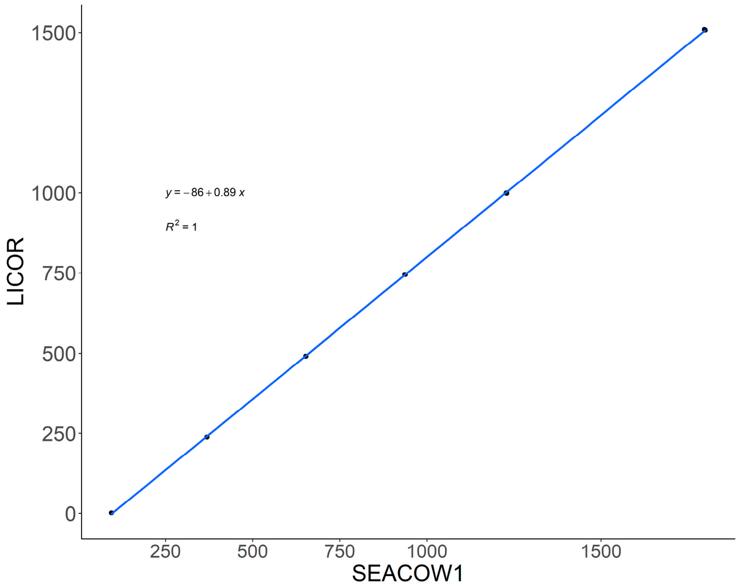

Figure S2. The average SEACOW1 pCO_2 (µatm) reading versus that of the LICOR LI-850 for a dry gas experiment described in Section 2.2.1. The linear regression is y = -86 + 0.89x with an R^2 = 1.

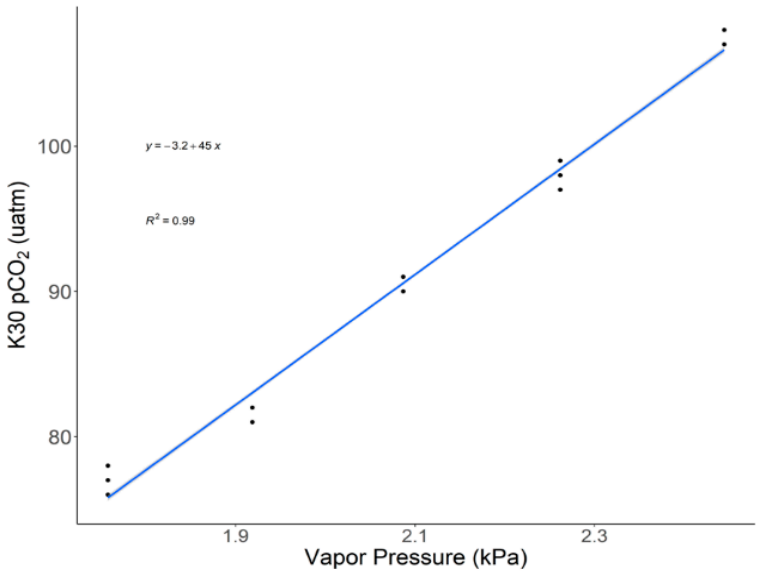

Figure S3. Linear relationship between vapor pressure (kPa) and K30 pCO_2 (µatm) readings: y = -3.2 +45x through temperatures 18-24 °C with an R^2 value of 0.99.

m_{dry} = 0.89; slope from SEACOW1's dry calibration curve (Figure S2)

b = -86 µatm; intercept from SEACOW1's dry calibration curve (Figure S2)

$K30_{raw}$ = 580 µatm; raw reading from K30

H = 57; percent of humidity provided by BME280 sensor

m_{H2O} = -3.2; slope from vapor pressure curve (Figure S3)

bH20 = 45 µatm; intercept from vapor pressure curve (Figure S3)

T = 20 °C = water temperature from TMP117 sensor

Therefore,

$V_{H2O} = 6 * 10^{-5}(20^3) + 5 * 10^{-4}(20^2) + 0.055(20) + 0.571 = 2.35$

So,

$K30_{H2O} = (-3.2 * 2.35 + 45) * (57/100) = 21.36$

So finally,

$K30_{CO2} = [(580 - 21.36) * 0.89] - 86 = 412$

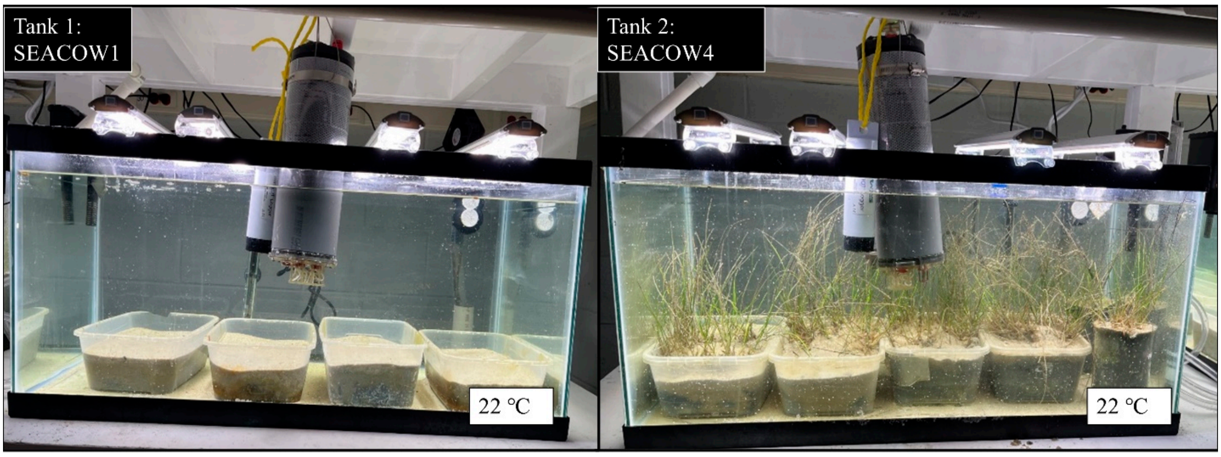

Figure S4. Experimental set up of the seagrass experiment. On the left is the control tank without seagrass, and on the right, the experiment tank with seagrass.

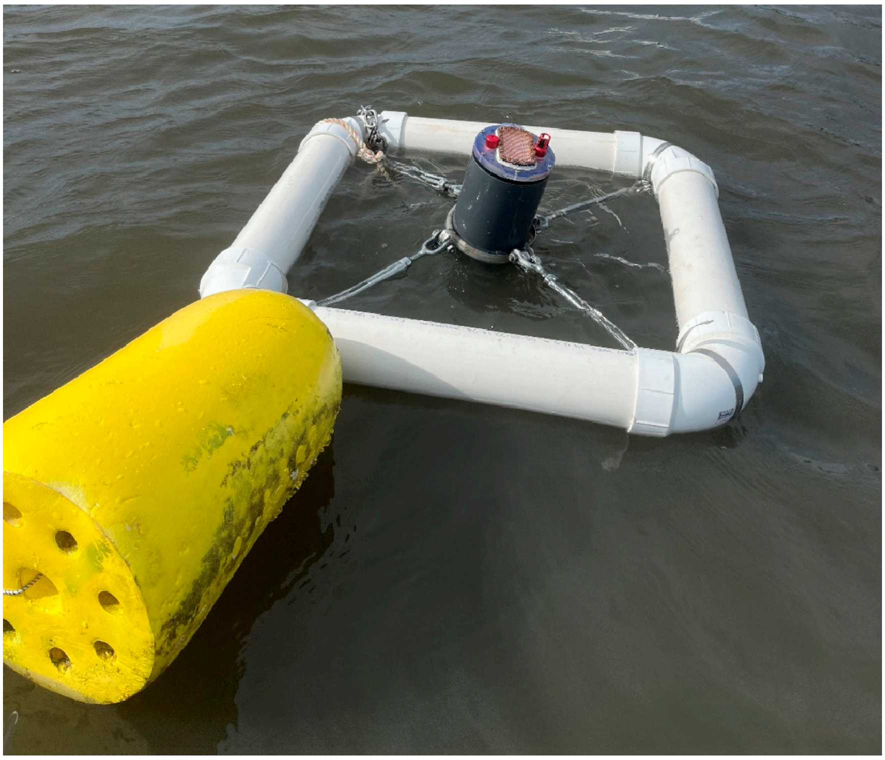

Figure S5. An example float made of PVC pipe to keep the SEACOW suspended at the air-water interface, made from 4 inch (10.16 cm) PVC, hose clamps, and turnbuckles. The entire float is approximately 2.5 x 2.5 feet (0.76 m x 0.76 m).
